# Comparative Evaluation of Nutrient Digestibility in Beagle Dogs of Different Life Stages

**DOI:** 10.3390/ani15202963

**Published:** 2025-10-13

**Authors:** Min Young Lee, Kyoung-Min So, Sang-Yeob Lee, Woo-Do Lee, Hyun-Woo Cho, Han Tae Bang, Seyeon Chang, Won Yong Jung, Kangmin Seo, Ju Lan Chun, Ki Hyun Kim

**Affiliations:** 1 Animal Welfare Division, National Institute of Animal Science, Rural Development Administration, Wanju 55365, Republic of Korea; 2 Ingredient Examination Division, Experiment Research Institute, National Agricultural Products Quality Management Service, Gimcheon 39660, Republic of Korea; 3 Academic-Industrial Cooperation Organization, Sunchon National University, Suncheon 57922, Republic of Korea

**Keywords:** nutrient, dog, life-stage, digestibility

## Abstract

**Simple Summary:**

This study compared nutrient digestibility across the life stages of dogs fed an identical diet. The digestibility of some major nutrients differed with age. Puppies exhibited reduced digestibility of crude protein and essential amino acids, whereas adults showed the highest ether extract digestibility. Phosphorus digestibility was highest in puppies and declined with age, whereas calcium digestibility did not differ. The digestibility values in seniors were slightly lower than those in adults; however, this difference was not statistically significant. These results highlight life stage-related differences in nutrient utilization and provide a basis for tailored dietary strategies in companion dogs.

**Abstract:**

This study evaluated age-related changes in nutrient digestibility in dogs and examined the effects of physiological development and dietary composition on digestive efficiency. Twenty Beagle dogs were assigned to three groups: puppies (<1 year; n = 8), adults (3–4 years; n = 8), and seniors (10–11 years; n = 4). All animals were fed diets formulated to contain identical nutrient levels that met or exceeded the minimum recommended nutrient requirements established by the Association of American Feed Control Officials. The digestibility of dry matter (DM), crude protein (CP), ether extract (EE), nitrogen-free extract (NFE), calcium (Ca), phosphorus (P), and amino acids were compared among the groups. The results showed that NFE digestibility was significantly higher in puppies, whereas CP digestibility was lower than that in adults and seniors, likely due to immature digestive function. In addition, EE digestibility was significantly lower in puppies, whereas P digestibility decreased with age. No significant difference was observed in Ca digestibility. Amino acid digestibility is lower in puppies, particularly for essential amino acids such as lysine, isoleucine, histidine, and arginine. These results indicate that age-related differences in digestive physiology and protein source affect nutrient utilization, providing a basis for developing life stage-specific nutritional strategies for companion animals.

## 1. Introduction

As companion animals are increasingly regarded as integral household members, there is a growing demand for scientifically grounded nutritional strategies that prioritize health and welfare [1,2]. This shift necessitates a departure from conventional feeding practices toward a more comprehensive approach that considers physiological characteristics and nutrient requirements across the lifespan [3,4,5].

Proteins, fats, carbohydrates, calcium (Ca), and phosphorus (P) are the major nutrients in companion animal diets, each playing distinct physiological roles essential for health, growth, and welfare throughout the lifespan [6]. Proteins are fundamental for tissue development, maintenance, and enzyme and hormone synthesis, whereas amino acids support musculoskeletal integrity and immune function [7,8]. Fat serves as a dense energy source, facilitates the absorption of fat-soluble vitamins, and contributes to cell membrane structure and neural function [9]. Carbohydrates provide readily available energy and act as substrates for the gut microbiota, thereby promoting intestinal health [10,11]. Ca is indispensable for bone and tooth formation, muscle contraction, and neural transmission, whereas P participates in bone metabolism with Ca and plays critical roles in cellular energy metabolism (ATP) and intracellular signaling [12].

For these nutrients to perform their normal physiological functions, it is not sufficient to simply meet their recommended minimum requirements. Their bioavailability, defined as the extent to which nutrients are digested, absorbed, and utilized in the body, must be ensured [13,14]. Age-related changes in gastrointestinal morphology, digestive enzyme activity, and gut microbial composition can markedly influence nutrient digestibility and utilization efficiency [15,16]. Therefore, a comprehensive evaluation of the digestibility and bioavailability of protein, fat, carbohydrates, Ca, and P is fundamental for establishing life stage-specific nutritional strategies [17]. This study investigated age-related differences in the digestibility of major nutrients in dogs, providing scientific evidence for tailored dietary formulations and life stage-based nutritional management.

## 2. Materials and Methods

### 2.1. Experimental Animals and Design

The objective of this study was to investigate age-related differences in digestibility; therefore, to control for breed-related variation, all dogs included were Beagles. This study was approved by the Institutional Animal Care and Use Committee (IACUC) of the National Institute of Animal Science, Republic of Korea (NIAS-2021-513). A total of 20 beagle dogs were used in this study, including eight puppies (four neutered males and four spayed females) aged 8–10 months (<1 year old), eight spayed adult dogs aged 3–4 years, and four spayed senior dogs aged 10–11 years. The experimental diet was provided to the dogs based on their individual metabolic energy requirements (MER, kcal/day = 132 × kg BW^0.75^), according to the recommendations of the National Research Council (NRC) [18]. For growing dogs in the puppy group, the MER was set to 1.2 times that of adult dogs. Dogs were fed once daily at the same time each day. All animals were housed individually in enclosures measuring 170 × 210 cm. The room temperature was maintained at 21 ± 1 °C with a relative humidity of 60 ± 10%. Water was provided ad libitum to the animals. According to the Association of American Feed Control Officials (AAFCO) [19] protocol, following a 7-day acclimation period to the test diets, total feces excreted over a 4-day collection period were collected and used to analyze apparent total tract digestibility (ATTD). Fecal samples were collected daily at 9:00 a.m., 1:00 p.m., and 5:00 p.m., throughout the study period. All collected fecal samples were stored below −18 °C until analysis. The dogs’ health was monitored daily and cared for by a veterinarian as needed.

### 2.2. Experimental Diets

In this study, a standardized diet was used to minimize variability and to enable controlled comparisons across life stages. The experimental diets were formulated to meet the minimum nutritional requirements for growing dogs, as specified by the AAFCO. The diet contained 31.84% crude protein (CP), 15.89% ether extract (EE), and 42.98% nitrogen-free extract (NFE), with a metabolizable energy (ME) of approximately 3970 kcal and a Ca-to-P (Ca/P) ratio of 1.22. Metabolizable energy (ME) was calculated based on the equation proposed by the NRC [20], using the following formula: ME (kcal/kg) = ([crude protein × 3.5] + [ether extract × 8.5] + [nitrogen-free extract × 3.5]) × 10. All ingredients in the experimental diet were available in powdered form from commercial sources, except for lard, and no flavoring agents or preservatives were included. The mixed ingredients were steam-cooked for 50 min, molded, cut into uniform pellets, dried at 60 °C for 40 min to standardize the moisture content, and stored at −4 °C. Before feeding, the diets were thawed at room temperature for 12 h and maintained at an ambient temperature. The chemical compositions of the experimental diet is presented in Table 1.

### 2.3. Sampling and Analysis

ATTD was determined using the total fecal collection method. Proximate analyses of the feed and fecal samples were conducted in accordance with the standard methods of the Association of Official Analytical Chemists.

ATTD was calculated using the following equation:

$$Apparent\;Total\;Tract\;Digestibility\;\left(ATTD,\%\right)=\frac{Nutrient\;intake-Nutrient\;in\;feces}{Nutrient\;intake}\times 100$$

Blood samples were collected from the jugular vein after a 12 h fasting period at the beginning and end of the experiment. Immediately after collection, blood was dispensed into EDTA-treated vacuum tubes (ref. 367861, BD Vacutainer, Franklin Lakes, NJ, USA). Whole blood in EDTA-treated tubes was used for complete blood count (CBC) analysis immediately after sampling, and CBC was measured using an automated hematology analyzer (IDEXX Laboratories, Inc., Westbrook, ME, USA). Fecal scoring was performed using the Bristol stool scale [21,22]. All fecal samples collected from the dogs were evaluated according to their morphological characteristics, and a single researcher conducted all assessments to maintain consistency.

### 2.4. Statistical Analysis

Statistical analyses were performed using R software version 4.4.1 (R Foundation for Statistical Computing, Vienna, Austria). As the sample sizes differed among groups and the assumptions of normality and homogeneity of variance were not met, a non-parametric Kruskal–Wallis test was conducted, followed by Dunn’s test for post hoc analysis. Statistical significance was set at *p* < 0.05 for all analyses.

## 3. Results

### 3.1. Physiological and Hematological Parameters

Table 2 presents the body weights and average daily feed intakes according to age. Body weight was lower in puppies than in adult and senior dogs. No feed residues were observed in any of the dogs, and the difference in average daily feed intake is considered to reflect the variation in body weight, as the feeding amount was adjusted based on individual body weight.

CBC analyses were performed to assess the dogs’ health status, to verify the safety of the diet during the experimental period, and to demonstrate that no abnormal clinical findings were observed. Although some inter-individual variations were observed, all values remained within the normal reference ranges, and veterinary clinical evaluations revealed no abnormal findings. The results of CBC analysis are presented in Table 3. In addition, fecal scores were consistent with Bristol stool chart types 3–4, indicating an ideal stool consistency.

### 3.2. ATTD of Nutrients by Age Group

The ATTD of the macronutrients is shown in Table 4. Dry matter (DM) digestibility did not differ significantly among the age groups (*p* > 0.05). In contrast, NFE digestibility differed significantly according to age (*p* < 0.05), with the puppy group exhibiting the highest digestibility (95%). The adult dog group exhibited a significantly lower value of 93.96%, whereas the senior dog group showed an intermediate value of 94.35%. CP digestibility differed significantly among the age groups (*p* < 0.001), with the puppy group (89.64%) showing significantly lower digestibility than that of the adult dog group (91.74%). EE digestibility differed significantly among age groups (*p* < 0.05), with the puppy (97.39%) and senior dog (97.47%) groups showing slightly lower digestibility than the adult dog group (97.84%). Ca digestibility did not differ significantly among the age groups. However, P digestibility showed a significant age-related difference (*p* < 0.01), with the puppy group exhibiting the highest digestibility at 36.12%, followed by the adult and senior dog groups at 32.94% and 30.71%, respectively.

Amino acid digestibility varied with age for several amino acids, including essential amino acids such as lysine, isoleucine, histidine, and arginine, as well as the non-essential amino acid alanine (Table 5). For most of these amino acids, the puppy group had the lowest digestibility, the adult dog group showed the highest digestibility, and the senior dog group showed intermediate digestibility values without significant differences between the other two groups. For example, lysine digestibility was 92.22%, 93.49%, and 93.01% in the puppy, adult dog, and senior dog groups, respectively. Similar patterns were observed for isoleucine (93.35%, 94.24%, 93.95%), histidine (92.13%, 93.21%, 93.01%), and alanine (92.08%, 93.03%, 92.44%). In contrast, arginine digestibility was significantly higher in the adult (95.96%) and senior (95.83%) dog groups than in the puppy group (95.01%).

## 4. Discussion

### 4.1. Physiological and Hematological Parameters

The relatively low body weight observed in puppies is attributable to their ongoing growth, whereas the higher body weight observed in older dogs may be explained by their individual characteristics. Meanwhile, the increase in body weight observed in the adult and senior groups during the experimental period may be attributed to the fact that the dogs were fed at a level of 132 kcal/kg^0.75^ of metabolic body weight, as suggested by Patil et al. [23]. However, maintenance energy requirements can vary depending on individual activity levels and physiological characteristics, and studies such as Finke and Rainbird et al. have reported lower maintenance energy requirements for laboratory Beagles [24,25]. Therefore, it is considered possible that the relatively higher energy allowance provided in this study contributed to the body weight gain observed in the adult and senior groups.

In addition, although some CBC parameters showed statistically significant differences among the age groups, all values remained within the normal reference ranges, and no clinical abnormalities were detected. Therefore, these variations are unlikely to have biological or clinical significance and can be interpreted as age-related physiological fluctuations within the healthy range.

### 4.2. Apparent Total Tract Digestibility of Macronutrients by Age Group

According to the results of this study, DM digestibility did not differ significantly among age groups. This finding suggests that DM digestibility, as an integrative indicator reflecting the overall digestibility of various nutrients such as CP, EE, and NFE, may have been influenced by compensatory effects among individual nutrients, thereby offsetting potential age-related differences in digestibility.

CP digestibility differed significantly according to age, with the puppy group exhibiting significantly lower values than the adult and senior dog groups. This difference could be attributed to variations in physiological developmental stages. Several studies have reported that puppies have lower nutrient digestibility than that of adult dogs. Gilham et al., Swanson et al., and Fahey et al. demonstrated that as dogs mature, the digestive organs become more developed, leading to improved digestibility of key nutrients [16,26,27]. Malo reported that glucose absorption in the small intestine increases from 9 weeks of age to adulthood. This increase is associated with greater expression of amino acid and carbohydrate transporters [28]. Weber et al. noted that gastric emptying time is shorter in younger animals, which may limit the contact time between digestive enzymes and chyme, thereby reducing nutrient absorption [15]. Although limited, some studies have investigated age-related changes in CP digestibility in dogs. Weber et al. compared digestibility at 11 and 60 weeks of age in four dog breeds: Miniature Poodle, Medium Schnauzer, Giant Schnauzer, and Great Dane, and reported significant improvements in CP digestibility across all breeds [15]. Comparable age-related trends in protein digestibility have been reported in other monogastric species, such as pigs. Wilson et al. showed that in piglets fed soybean protein between 7 and 35 days of age, amino acid digestibility improved with age, suggesting that hindgut fermentation beyond the ileum may influence digestibility measurements [29]. Similarly, Engelsmann et al. reported that the standardized ileal digestibility of CP and amino acids increased over time in weaned piglets fed various protein sources (wheat, soybean meal, enzyme-treated soybean meal, hydrothermally treated rapeseed meal, and casein) [30]. Overall, these findings suggest that, although growing animals have higher protein requirements than adults, their relatively lower digestive efficiency may limit the actual nutrient bioavailability. Therefore, it is crucial to select protein sources with high digestibility when formulating diets for these growth stages. Considering age-specific digestive physiology is essential for appropriate ingredient selection. Thus, considering the digestive physiology across different life stages is essential for the appropriate selection of dietary ingredients. Furthermore, as variations in ingredient composition or nutrient levels may affect digestibility at different life stages, further research in this area is warranted. In addition, integration with advanced approaches such as the ileal digestibility method, metabolomics or gut microbiota analysis could provide a more precise understanding of nutrient digestion and utilization. Such an approach is expected not only to clarify age-specific nutritional requirements but also to strengthen the scientific basis for future companion animal diet formulation.

Previous studies have reported inconsistent findings regarding EE digestibility. Sabchuk et al. and Domingues did not observe any significant age-related differences [17,31]. In contrast, Félix et al. reported higher fat digestibility in puppies than in adults in a study involving dogs of similar age ranges to those in the current study (5.1 ± 0.2 months and 5.8 ± 0.1 years). The authors explained that this may have been due to the relatively higher apparent metabolizable energy intake per unit body weight in puppies, which could have led to an overestimation of fat digestibility [32]. Additionally, Zanatta et al. reported that dogs older than 5 months had higher fat digestibility than 3-month-old puppies in a study involving animals at various developmental stages (3, 5, 9, and 15 months) [33]. These inconsistent findings may be attributable to differences in breed, growth stage, and diet nutrient composition. In contrast, the current study observed that the puppy group exhibited significantly lower EE digestibility than the adult dog group. This result contradicts previous findings. This discrepancy may be related to differences in the physiological digestive development, energy intake, and metabolic efficiency of the growing puppies. Therefore, the findings of this study suggest that the selection of fat sources and diet formulations should consider age-specific digestive and physiological characteristics.

The digestibility of NFE differed significantly among age groups, with pups exhibiting relatively higher values. As NFE is a calculated parameter derived from the digestibility of other nutrients rather than a directly measured component, these results may reflect indirect effects associated with changes in the digestibility of other macronutrients.

### 4.3. Apparent Total Tract Digestibility of Ca and P by Age Group

Ca digestibility showed variable patterns depending on age. Ca is absorbed through both passive and active mechanisms; however, in growing puppies, the passive pathway is believed to play a more prominent role because of their increased Ca requirements [16]. However, no significant differences in Ca digestibility were observed among the age groups in the current study. This may be attributed to the fact that the puppies used in the experiment were 8–10 months old, an age beyond the rapid growth phase, during which Ca utilization is typically highest.

The digestibility of P differed significantly with age. P is the second-most abundant mineral in the body after Ca and plays various physiological roles beyond bone formation, including energy metabolism and intracellular signaling [34,35]. However, few studies have investigated age-related changes in P metabolism [16]. In a previous study conducted in pigs, aging decreased the secretion and absorption efficiency of endogenous P in the gastrointestinal tract [36]. Similarly, Vorland et al. demonstrated that intestinal P absorption efficiency was higher in growing rats, with a decline observed as age increased, owing to reduced metabolic demand [37]. Consistent with these findings, the current study observed significantly higher P digestibility in juvenile dogs than in adult or senior dogs. This may be attributed to the elevated P requirements and more active bone metabolism in growing individuals, leading to more efficient digestion and absorption of P. Furthermore, P digestibility can be influenced by various factors, including interactions with Ca [38,39], the presence of binding compounds such as phytate [40,41], and gut microbiota composition [42]. Owing to these complex interacting factors, mineral digestibility tends to exhibit high interindividual variability, as reflected by the relatively large standard error observed in this study. Therefore, future studies should adopt a multifaceted approach that includes inorganic and organic P fractionation, quantification of phytate P (phytate-P) binding, and 16S rRNA-based analysis of gut microbial communities. These approaches are expected to enhance the precision of digestibility assessments and improve the overall reliability and interpretability of the results.

### 4.4. Apparent Total Tract Digestibility of Amino Acids by Age Group

Similarly to CP digestibility, the digestibility of most amino acids was lower in puppies, with significant reductions observed in essential amino acids such as lysine, isoleucine, histidine, and arginine [43]. Since essential amino acids cannot be synthesized endogenously, they must be supplied through the diet, where they play pivotal roles in muscle and tissue protein synthesis, the production of enzymes and hormones, and the activation of immune cells, thereby supporting growth, development, and immune function [44,45]. Consequently, reduced efficiency in the digestion and absorption of essential amino acids during the puppy stage may lead to physiological disadvantages, including impaired protein synthesis, growth retardation, and weakened immune responses [46,47,48,49]. For this reason, it is critical to select protein sources with high digestibility and a well-balanced essential amino acid profile when formulating puppy diets, and to establish nutritional strategies tailored to the unique physiological demands of the growth stage.

### 4.5. Effects of Aging on Nutrient Digestibility in Senior Dogs

In the current study, the senior dog groups showed slightly lower digestibility of most nutrients than the adult dog groups; however, these differences were not statistically significant. Aging is defined as a gradual decline in the function of various organ systems after physiological maturity [50]. Factors such as impaired gastrointestinal function, altered transit time, and reduced gastric acid and bile secretion have been identified as potential causes [51,52]. Handler et al. and Burkholder reported that reduced secretion of pancreatic enzymes and bile acids in older dogs may lead to decreased fat digestibility [53,54]. However, there is limited consistent evidence that aging directly results in reduced nutrient digestibility, although some studies have reported that older dogs maintain or improve digestibility [52,55]. This study has the limitation that the small number of senior dogs may have reduced the statistical power; however, the findings are consistent with previous reports showing no decline in digestibility in senior dogs, suggesting that physiological aging may not necessarily lead to a reduction in nutrient digestibility in older dogs. This supports the rationale behind the current nutritional guidelines of the NRC and European Pet Food Industry Federation (FEDIAF), which do not differentiate between adult and senior dogs based on their nutritional recommendations [19,56]. Further research involving larger sample sizes and a broader range of breeds is considered necessary to establish this more conclusively.

## 5. Conclusions

This study compared and analyzed the changes in nutrient digestibility associated with growth and aging in dogs, confirming that age-related differences in digestive physiology can influence the bioavailability of the dietary nutrients. As a result, puppies exhibited distinct differences in nutrient digestibility compared with adult and senior dogs, suggesting that the digestive physiological characteristics during the growth stage play an important role in nutritional management. In contrast, adult and senior dogs showed similar digestibility, indicating that a consistent nutritional management strategy can be applied to these two life stages. Overall, the findings provide fundamental scientific evidence supporting the necessity of life stage-specific nutritional management in companion dogs.

## Figures and Tables

**Table 1:** Analyzed chemical composition of experimental diets.

Item (%)	Chemical Composition (% [DM Basis], Analysis)
**CP**	31.84
**EE**	15.89
**CF**	1.41
**CA**	7.87
**NFE**	42.98
**Ca**	1.69
**P**	1.23
**Ca/P**	1.22
**ME** (kcal/kg)	3969.35
**Amino Acid**	
Lys	2.03
Met	0.78
Thr	1.41
Val	1.65
Leu	2.53
Ile	1.40
Phe	1.41
His	0.81
Arg	1.93
Trp	0.22

CP, crude protein; EE, ether extract; CF, crude fiber; CA, crude ash; NFE, nitrogen-free extract; Ca, calcium; P, phosphorus; Ca/P, calcium to phosphorus ratio; Lys, lysine; ME, Metabolizable energy; Met, methionine; Thr, threonine; Val; Valine; Leu, leucine; Ile, isoleucine; Phe, phenylalanine; His, histidine; Arg, arginine; Trp, tryptophan; NFE was calculated using the following equation: NFE = Dry matter − (Crude protein + Ether extract + Crude ash).

**Table 2:** Body weight and average daily feed intake of dogs by age group.

	Puppy	Adult Dog	Senior Dog	SEM	*p* Value
**MEI** (kcal/day)	957.44 ^a^	857.36 ^b^	1157.82 ^c^	29.88	<0.001
**Body weight** (kg)					
Initial	7.03 ^a^	11.59 ^b^	17.76 ^c^	0.99	<0.001
Final	8.35 ^a^	12.36 ^b^	18.47 ^c^	0.41	<0.001
**ADI** (g, [DM basis])	241.21 ^a^	216.00 ^b^	291.70 ^c^	6.13	<0.01
**BWG** (g)	1320.00	1271.25	1500.00	0.16	0.886

Values are reported as means (puppy group, n = 8; adult group, n = 8; senior group, n = 4); values in the same row with different superscript letters do not differ significantly (*p* < 0.05). MEI, Metabolizable energy intake; ADI, Average daily feed intake; BWG, Body weight gain; SEM, standard error of the mean.

**Table 3:** Complete blood count (CBC) parameters in dogs across age groups.

Item	UNIT	Ref. Range	Puppy	Adult Dog	Senior Dog	SEM	***p* Value**
**WBC**	10^3^/µL	6.00–17.00	9.29 ^a^	7.25 ^b^	8.07 ^ab^	0.243	<0.001
**NEU**	10^3^/µL	3.62–12.30	5.82 ^a^	4.60 ^b^	5.26 ^a^	0.210	<0.01
**LYM**	10^3^/µL	0.83–4.91	2.91 ^a^	2.06 ^b^	2.01 ^b^	0.066	<0.001
**MON**	10^3^/µL	0.14–1.97	0.42 ^a^	0.29 ^b^	0.48 ^a^	0.018	<0.001
**EOS**	10^3^/µL	0.04–1.62	0.10 ^a^	0.29 ^b^	0.31 ^b^	0.018	<0.001
**BAS**	10^3^/µL	0.00–0.12	0.04 ^a^	0.01 ^b^	0.01 ^b^	0.002	<0.001
**NEU%**	%	52.00–81.00	61.20	62.02	64.43	0.703	0.186
**LYM%**	%	12.00–33.00	32.82 ^a^	29.66 ^ab^	25.68 ^b^	0.710	<0.001
**MON%**	%	2.00–13.00	4.34 ^a^	4.13 ^a^	6.06 ^b^	0.164	<0.001
**EOS%**	%	0.50–10.00	1.17 ^a^	4.05 ^b^	3.70 ^b^	0.226	<0.001
**BAS%**	%	0.00–1.30	0.47 ^a^	0.14 ^b^	0.13 ^b^	0.022	<0.001
**RBC**	10^6^/µL	5.10–8.50	6.92 ^a^	7.71 ^b^	6.46 ^c^	0.065	<0.001
**HGB**	g/dL	11.00–19.00	16.58 ^a^	17.65 ^b^	14.96 ^c^	0.137	<0.001
**HCT**	%	33.00–56.00	44.95 ^a^	48.51 ^b^	42.23 ^c^	0.358	<0.001
**MCV**	fL	60.00–76.00	64.98 ^a^	62.94 ^b^	65.54 ^a^	0.208	<0.001
**MCH**	Pg	20.00–27.00	24.04 ^a^	22.91 ^b^	23.20 ^b^	0.128	<0.001
**MCHC**	g/dL	30.00–38.00	37.00 ^a^	36.40 ^a^	35.42 ^b^	0.183	<0.001
**RDW-CD**	%	12.50–17.20	13.48 ^a^	13.29 ^a^	12.91 ^b^	0.059	<0.01
**RDW-SD**	fL	33.20–46.30	35.12 ^a^	33.36 ^b^	33.95 ^b^	0.183	<0.001
**PLT**	10^3^/µL	117.00–490.00	287.54 ^a^	342.92 ^b^	419.75 ^c^	8.701	<0.001
**MPV**	fL	8.00–14.10	9.88 ^a^	10.01 ^a^	10.87 ^b^	0.089	<0.01
**PDW**		12.00–17.50	15.28 ^a^	15.22 ^a^	15.73 ^b^	0.029	<0.001
**PCT**	mL/L	0.90–5.80	2.84 ^a^	3.40 ^b^	4.48 ^c^	0.087	<0.001

Values are expressed as means (puppy group, n = 8; adult group, n = 8; senior group, n = 4); values in the same row with different superscript letters do not differ significantly (*p* < 0.05). WBC, white blood cell count; NEU, neutrophils; LYM, lymphocytes; MON, monocytes; EOS, eosinophils; BAS, basophils; NEU%, percentage of neutrophils; LYM%, percentage of lymphocytes; MON%, percentage of monocytes; EOS%, percentage of eosinophils; BAS%, percentage of basophils; RBC, red blood cell count; HGB, hemoglobin; HCT, hematocrit; MCV, mean corpuscular volume; MCH, mean corpuscular hemoglobin; MCHC, mean corpuscular hemoglobin concentration; RDW-CD, red cell distribution width–coefficient of deviation; RDW-SD, red cell distribution width–standard deviation; PLT, platelet count; MPV, mean platelet volume; PDW, platelet distribution width; PCT, plateletcrit.

**Table 4:** Apparent total tract digestibility of macronutrients (%).

	Puppy	Adult Dog	Senior Dog	SEM	*p* Value
**DM**	88.17	88.89	87.46	0.21	0.286
**NFE**	95.00 ^a^	93.96 ^b^	94.35 ^ab^	0.18	<0.01
**CP**	89.64 ^a^	91.74 ^b^	90.95 ^ab^	0.26	<0.001
**EE**	97.39 ^a^	97.84 ^b^	97.47 ^a^	0.09	<0.05
**Ca**	14.58	17.29	12.05	1.71	0.672
**P**	36.12 ^a^	32.94 ^ab^	30.71^b^	0.83	<0.05

Values are expressed as means (puppy group, n = 8; adult group, n = 8; senior group, n = 4); values in the same row with different superscript letters do not differ significantly (*p* < 0.05). DM, dry matter; NFE, nitrogen-free extract; CP, crude protein; EE, ether extract; Ca, calcium; P, phosphorus; SEM, standard error of the mean.

**Table 5:** Apparent total tract digestibility of amino acids (%).

	Puppy	Adult Dog	Senior Dog	SEM	*p* Value
**Essential amino acid**					
Lys	92.22 ^a^	93.49 ^b^	93.01 ^ab^	0.18	<0.01
Met	92.93	93.40	93.15	0.24	0.154
Thr	91.99	92.54	92.28	0.16	0.287
Val	92.45	90.81	90.56	0.36	0.986
Leu	94.02	94.51	94.22	0.14	0.374
Ile	93.35 ^a^	94.24 ^b^	93.95 ^ab^	0.16	<0.05
Phe	93.50	93.88	93.56	0.14	0.504
His	92.13 ^a^	93.21 ^b^	93.01 ^ab^	0.16	<0.05
Arg	95.01 ^a^	95.96 ^b^	95.83 ^b^	0.10	<0.001
**Non-essential Amino acid**					
Asp	91.69	92.45	91.79	0.16	0.147
Glu	93.32	93.91	93.37	0.15	0.167
Ser	85.42	86.33	85.82	0.18	0.143
Tyr	93.34	92.89	92.64	0.15	0.049
Cys	84.43	84.89	83.40	0.37	0.094
Pro	92.75	93.14	92.89	0.14	0.636
Gly	89.64	90.66	90.17	0.27	0.224
Ala	92.08 ^a^	93.03 ^b^	92.44 ^ab^	0.18	<0.05

Values are expressed as means (puppy group, n = 8; adult group, n = 8; senior group, n = 4); values in the same row with different superscript letters do not differ significantly (*p* < 0.05). Lys, lysine; Met, methionine; Thr, threonine; Val, valine; Leu, leucine; Ile, isoleucine; Phe, phenylalanine; His, histidine; Arg, arginine; Asp, aspartic acid; Glu, glutamic acid; Ser, serine; Tyr, tyrosine; Cys, cysteine; Pro, proline; Gly, glycine; Ala, alanine. SEM, standard error of the mean.

## Data Availability

The original contributions presented in this study are included in the article. Further inquiries can be directed to the corresponding author.
